# Dissemination of Information to Foreigners in Preparation for Natural Disasters: Response to a Letter to the Editor

**DOI:** 10.31662/jmaj.2024-0360

**Published:** 2025-01-31

**Authors:** Akiko Morimoto, Nao Sonoda

**Affiliations:** 1 Graduate School of Nursing, Osaka Metropolitan University, Osaka, Japan

**Keywords:** preparedness, earthquake, international student, Japan

We thank Kido et al. ^(1)^ for reading our previous manuscript in the Journal entitled “Individual Preparedness for Large-scale Earthquakes among International Students in Japan: A Cross-sectional Questionnaire Survey” ^(2)^ and for providing valuable comments on dissemination of information to foreigners. The study showed that many international students in Japan were not prepared for large-scale earthquakes ^(2)^. Here, we are providing responses to address your comments.

First, we agree with you on the need to promote “plain Japanese” in disaster communications. Currently, some municipalities provide information on individual preparedness for earthquakes in plain Japanese on their websites ^(3)^. Glossaries are also available ^(4)^. We believe that it is necessary to spread information on individual preparedness for earthquakes in plain Japanese, mainly by municipalities.

Second, we agree with you on the need to promote multilingual disaster communication. In our study ^(2)^, the international students came from a diverse range of countries, including China, Vietnam, Myanmar, Nepal, Hong Kong, Taiwan, Thailand, Bangladesh, India, Sri Lanka, the United States, Russia, and Mexico. Moreover, native language (68.4%) was the most common language used when gathering information on individual preparedness for large-scale earthquakes, followed by Japanese (64.9%) and English (36.0%) ^(2)^. Therefore, we believe that it is important to promote multilingual disaster communications by utilizing machine translation tools, as suggested by Kido et al. ^(1)^.

Third, we agree to provide printouts with critical phrases in Japanese and the individual’s native language. Although we believe that websites in plain Japanese and machine translation tools are useful, it is estimated that the Nankai Trough Earthquake will cause 5.8 million communication failures ^(5)^. Therefore, it is important to provide printouts containing critical phrases in case of communication failure.

Foreign residents, including international students, in Japan are considered more vulnerable to serious disasters because of limited information because of language barriers. To promote their preparedness for large-scale earthquakes, further studies are needed on how to provide information.

## Article Information

### Conflicts of Interest

None

### Author Contributions

Both authors wrote and revised the paper, and AM had primary responsibility for the final content. Both authors read and approved the final manuscript.

### Approval by Institutional Review Board (IRB)

Not applicable
